# A Case of Silicone and Sarcoid Granulomas in a Patient with “Highly Cohesive” Silicone Breast Implants: A Histopathologic and Laser Raman Microprobe Analysis

**DOI:** 10.3390/ijerph18094526

**Published:** 2021-04-24

**Authors:** Todor I. Todorov, Erik de Bakker, Diane Smith, Lisette C. Langenberg, Linda A. Murakata, Mark H. H. Kramer, Jose A. Centeno, Prabath W. B. Nanayakkara

**Affiliations:** 1 Department of Environmental and Infectious Disease Sciences, Division of Biophysical Toxicology, Armed Forces Institute of Pathology, Washington, DC 20306, USA; todor.todorov@fda.hhs.gov (T.I.T.); lmurak@aol.com (L.A.M.); jacenteno@comcast.net (J.A.C.); 2 Department of Plastic Surgery, VU University Medical Centre, P.O. Box 7057, 1007 MB Amsterdam, The Netherlands; e.debakker@amsterdamumc.nl; 3 Department of Molecular Cell Biology and Immunology, VU University Medical Centre, P.O. Box 7057, 1007 MB Amsterdam, The Netherlands; 4 Henry Jackson Foundation, Bethesda, MD 20817, USA; Diane.Smith@fda.hhs.gov; 5 Department of Internal Medicine, VU University Medical Centre, P.O. Box 7057, 1007 MB Amsterdam, The Netherlands; lisette.langenberg@gmail.com; 6 Department of Pathology, VU University Medical Centre, P.O. Box 7057, 1007 MB Amsterdam, The Netherlands; M.kramer@amsterdamumc.nl

**Keywords:** breast implant, silicone, sarcoidosis, Raman, Schaumann bodies

## Abstract

Foreign body giant cell (FBGC) reaction to silicone material in the lymph nodes of patients with silicone breast implants has been documented in the literature, with a number of case reports dating back to 1978. Many of these case reports describe histologic features of silicone lymphadenopathy in regional lymph nodes from patients with multiple sets of different types of implants, including single lumen smooth surface gel, single lumen textured surface gel, single lumen with polyethylene terephthalate patch, single lumen with polyurethane coating, and double lumen smooth surface. Only one other case report described a patient with highly-cohesive breast implants and silicone granulomas of the skin. In this article, we describe a patient with a clinical presentation of systemic sarcoidosis following highly cohesive breast implant placement. Histopathologic analysis and Confocal Laser Raman Microprobe (CLRM) examination were used to confirm the presence of silicone in the axillary lymph node and capsular tissues. This is the first report where chemical spectroscopic mapping has been used to establish and identify the coexistence of Schaumann bodies, consisting of calcium oxalate and calcium phosphate minerals, together with silicone implant material.

## 1. Introduction

Silicone gel breast implants have been subject to controversies since their introduction by Cronin and Gerow in the early 1960s [1]. The safety of these implants first came into question after several reports were published on a possible association of silicone breast implants and development of connective tissue disease in the 1980s [2,3,4,5,6]. Other possible adverse effects of silicone implants include capsular contracture, rupture [7], locoregional complications, silicone migration to the lung, skin and lower extremities [8,9], and interference with cancer detection [10]. To reduce these probable adverse effects, “highly cohesive” implants were introduced into the market in 1994. The cohesiveness of the silicone [polydimethylsiloxane (PDMS)] polymer and a specially designed outer membrane in these implants were developed to reduce the incidence of rupture and gel bleeds, characterized by gel leakage through the shell of the implant [11,12,13]. Several authors reported systemic responses in patients with silicone breast implants, including PDMS prostheses [13,14,15]. We now report the first case of a patient with highly cohesive breast implants who exhibited a sarcoidosis-like clinical presentation and silicone-containing granulomas at a distant site from her breasts. This case is especially enlightening in the context of the current attention around a number of other reported adverse effects (arthralgia, myalgia, fatigue, pyrexia, sicca) related to breast implants [16]. Therefore, we acknowledge the importance of sharing this account in the present climate of the enhanced understanding regarding exposure to such materials.

### Case Report

A 40-year-old Nigerian born woman, living in the Netherlands since 1985, underwent bilateral breast augmentation in 1996. The implants that she received were the highly-cohesive silicone gel implants (Style-410) (Inamed aesthetics, formerly McGhan Medical, Santa Barbara, CA, USA). Postoperatively, no complications were reported, except for occasional short-lived episodes of breast hardening.

In July 2001, she developed stone-hard tender breasts, which were predominantly on the right side. A few weeks later she noticed multiple firm, non-tender skin nodules that were located between the breasts and under the right breast. The nodules were approximately 0.5 cm in diameter, and after several days they began to ulcerate and produced a whitish fluid. She was seen by her plastic surgeon who concluded that she had eczema. In the following eight months, the ulcerations and symptoms worsened, and the implants had to be surgically removed. During the operation the surgeon noted that the fibrous capsule surrounding the implant was severely contracted. A biopsy was taken from the lymph node located in the left axillary and cultures were sent for fungi and mycobacteria. Macroscopic examination of the breast explants showed no obvious leakage. PCR for mycobacteria was negative and cultures for fungi and bacteria were also negative.

Six months after the removal of the breast implants, the patient presented to the internal medicine outpatient clinic with an increasing number of nodules scattered all over her body. Her eyes and mouth were dry, her appetite reduced, she lost 2 kg of weight over two months and she complained of extreme lassitude. She had no fever or night sweats but had slight dyspnea on exertion. The patient denied a history of intravenous drug use or liquid silicone injections. On physical examination, she had firm, non-tender skin nodules about 1 cm in diameter on her face (upper eyelids, nose, cheeks), arms, and legs. The nodules were moveable and did not appear to be attached to the skin surface or underlying structures. Both breasts were hard and tender on palpation. In addition, she had swelling in both her upper eyelids, which became so severe that she could barely open her eyes. She also had a moderate visual impairment in her left eye and ophthalmological examination showed features of panuveitis. There was generalized lymphadenopathy. The axillary and inguinal lymph nodes were firm, non-tender, and approximately 2 cm in diameter, while a left supraclavicular lymph node was about 1.5 cm in diameter. Other physical findings included normal heart and lung sounds, mild hepatomegaly and the spleen was not palpable.

A chest x-ray and CT scan of the thorax showed multiple enlarged lymph nodes in the hilum and axilla bilaterally, and fine nodular interstitial infiltrates in her lungs. Her sedimentation rate was 23 mm/h (normal < 10 mm/h), c-reactive protein 25 mg/L(normal < 8 mg/L), Hb 7.8 mmol/L (normal 7.5–10 mmol/L), WBC count 4.7 × 109/L (normal 3–10 × 10^9^/L), creatinine 88 μmol/L (normal 60–110 μmol/L), gamma glutamyltransferase 201 µ/L (normal 10–50 µ/L), alanine aminotransferase 66 µ/L (normal 4–36 µ/L), and the angiotensin converting enzyme (ACE) level was 63 µ/L (normal < 25 µ/L).

The patient was diagnosed with sarcoidosis. No prior family history of sarcoidosis was noted, and the patient did not have a history of autoimmune or autoinflammatory disease. She refused to take prednisone and was treated with minocycline, an antibiotic with presumed anti-granulomatous activity, 100 mg twice per day for 7 months with moderate improvement in her condition. Minocycline has also been shown to have autoimmune and anti-inflammatory diseases [17]. She did not develop any new nodules and the existing nodules became smaller. She also noted a decrease in the amount of dryness of her eyes and mouth.

## 2. Materials and Methods

### 2.1. Histology

Biopsies were taken from the fibrous capsule surrounding the implants, a lymph node in the left axillary, a nodule on the left lower leg and a nodule on the eyelid. For each type of tissue, sections 4–6 µm in thickness were stained with hematoxylin and eosin (HE) and evaluated using ordinary light microscopy. The presence or absence of the following histologic features were evaluated: (1) silicone-induced granuloma reaction; (2) sarcoid-like granulomas; (3) giant cells; and (4) refractile material consistent with silicone. Sections (2–6 µm thick) were also prepared for the CLRM experiments.

Additional case material consisted of the implant removed from the patient, herein referred to as the “explant.” The explant was received and maintained without addition of fixative solutions. The explant material was examined using CLRM.

### 2.2. Chemical Microspectroscopy

CLRM measurements were conducted as published elsewhere [18,19]. Briefly, CLRM experiments were performed on sections prepared from capsular tissue, axillary lymph node, eyelid nodule, and leg nodule using a LabRAM spectrograph (Jobin Yvon Horiba and Dilor, Palaiseau, France). The instrument is equipped with a He:Ne laser having an excitation wavelength at 632 nm. The spectrograph is interfaced to a high stability BX 40 Olympus microscope equipped with objectives at 10×, 50× and 100×. Laser spot diameters of approximately 2 and 1 µm could be obtained using the 50 and 100× objectives, respectively. The 100× objective was used to focus the laser beam onto the sample and to collect the Raman spectral images. The microscope system is also equipped with an adjustable confocal hole ranging from 100–1000 µm aperture size, allowing for reduction on stray light and the removal of unwanted laser plasma lines. Once the laser is focused onto the sample using the 100× objective, the scattered light is collected using the same objective, collimated and focused on the entrance slit of the spectrograph. The spectrometer was equipped with two gratings mounted on the same shaft blazed at 1800 grooves/nm (holographic) and 950 grooves/nm, respectively. The Raman signal is then measured with a detection system consisting of a charge-couple detector with an active detector window of 1024 × 256 pixels. The viewing area containing the foreign materials was approximately 5 × 5µm. Each spectrum was the result of 12 scans, at a spectral resolution of 4 cm^−1^.

Raman micro spectroscopy chemical finger printing has been used to identify silicone migration away from the implant [18,19,20,21]. When incident coherent light interacts with molecule, much of the light scatters with the same frequency as that of the incident light, referred to as elastic scattering. A small portion of the scattered photons have a frequency greater or less than that of the incident light, known as inelastic scattering. The difference is equal to the vibrational frequency of the molecular bond with which the photons interacted. The frequency of this scattered light contains information that can be correlated to the molecular species that led to the frequency shift [20]. This frequency shift arises from polarizable molecules, or those that have an inducible dipole moment. This phenomenon was first characterized by C.V. Raman and has been developed into a characterization tool that can be used to identify chemical signatures of organic and inorganic material relevant to the present investigation [22,23,24].

In this study, we have used the mapping capabilities of the Raman microprobe system to study the multi-compositional characteristics of foreign inclusions associated with breast implants and mineral deposition, allowing us to simultaneously identify silicone, calcium oxalate and other compounds associated with the appearance of Schaumann bodies.

## 3. Results

### 3.1. Histology

#### 3.1.1. Capsule Surrounding the Implant, Bilateral

**Figure 1:**
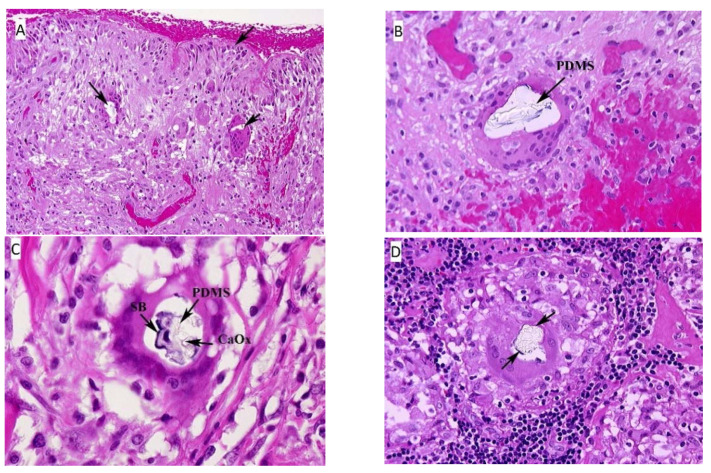
(**A**) Fibrous capsule surrounding the implant, right. The surface of the tissue lies adjacent to the silicone breast implant and forms the “pseudosynnovium.” This pseudosynnovium is composed of amorphous eosinophilic material and mononuclear cells. Of note are several foreign body giant cells containing clear silicone gel. There is a chronic inflammatory infiltrate scattered among the fibroblasts, fibrous tissue, and collagen. (HE, 10×). (**B**) Fibrous capsule surrounding implant, right. A high-power view of the above tissue shows silicone gel within the giant cell that is embedded in fibrous tissue. There are also scattered chronic inflammatory cells, congested blood vessels, and surgical related hemorrhage. (HE, 100×). (**C**) Fibrous capsule surrounding implant, left. High power view of previous section. In the center of the multinucleated giant cell are fragments of Schaumann body (annotated on the image as SB), and refractile clear crystalline material consistent with calcium oxalate (annotated on the image as CaOx). (HE, 100×). (**D**) Axillary lymph node, left. There is clear refractile globular material in the center of the giant cell of the epithelioid granuloma consistent with silicone gel. In addition, fragments of a Schaumann body can be seen around the outer edge of the silicone gel. (HE, 100×). Arrows on the HE images, unless designated point towards areas of interest such as migrated PDMS.

Hematoxylin-eosin (HE) stained sections showed a thick fibrous capsular wall with an inner lining of amorphous eosinophilic material, mononuclear cells and congested blood vessels forming a “pseudosynovium” (Figure 1A) [25]. There were numerous non-caseating granulomas, foreign body and Touton-type giant cells, and scattered chronic inflammatory cells within fibrous tissue. The granulomas were more prominent in the mid-section of the capsule wall, and their composition varied from a few epithelioid cells to large nodular aggregates surrounded by a thick fibrous collar. The foreign-body and Touton-type giant cells contained vacuoles with occasional Schaumann bodies, birefringent crystalline material, and clear refractile, non-birefringent material. The clear refractile material was confirmed as PDMS (Figure 1B) by confocal Raman microprobe analysis. Occasionally, all three of the inclusions were seen in the same giant cell (Figure 1C).

#### 3.1.2. Axillary Lymph Node, Left

The lymph nodes were almost totally replaced by non-caseating epithelioid granu- lomas of varying sizes and contained the same type of PDMS-based inclusions as seen in the fibrous capsule surrounding the implants (Figure 1D). Granulomas were also seen in the lymph node hilum.

#### 3.1.3. Eyelid Nodule

**Figure 2:**
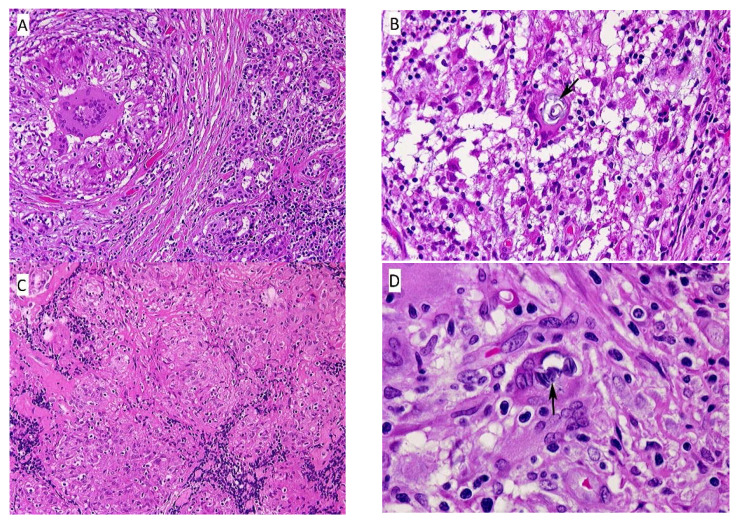
(**A**) Eyelid nodule, left. This section contains both normal serous glands of the eyelid (right), and fibrous tissue surrounding a non-caseating granuloma with central foreign body giant. (HE, 10×); (**B**) Eyelid nodule, left. This section shows a non-caseating granuloma with a Schaumann body within the foreign body giant (center). (HE, 100×); (**C**) Lower leg nodule, left. There are aggregates of varying-sized epithelioid granulomas, fibrous tissue septae, and scattered lymphocytes. (HE, 10×); (**D**) Lower leg nodule, left. The section contains a non-caseating granuloma with scattered lymphocytes and several multinucleated giant cells in a fibrous tissue background. The giant cell in the center, indicated by the black arrow, contains two Schaumann bodies. (HE, 100×).

The biopsy showed multiple epithelioid granulomas, septa of dense fibrous tissue, and lobules of serous (lacrimal) glands with focal lymphocytic infiltration (Figure 2A). In some foci there were degenerating ducts and glands admixed with chronic inflammation. Many of the epithelioid cells had clearing of the cytoplasm (“clear-cells”), and a rare Schaumann body was seen (Figure 2B). One small focus of necrosis was found in one granuloma; however, special stains were negative for fungi and bacteria.

#### 3.1.4. Leg Nodule

The majority of tissue was replaced by sheets of non-caseating granulomas (Figure 2C), histologically typical of sarcoidosis. Some smaller nodules were surrounded by fibrous tissue with thick fibrous septae and contained varying amounts of chronic inflammatory cells. Multinucleated, foreign-body giant cells with an occasional Schaumann body were present, as well as scattered lymphocytes, plasma cells, and occasional eosinophils and neutrophils (Figure 2D).

### 3.2. Confocal Laser Raman Microscopy

**Figure 3:**
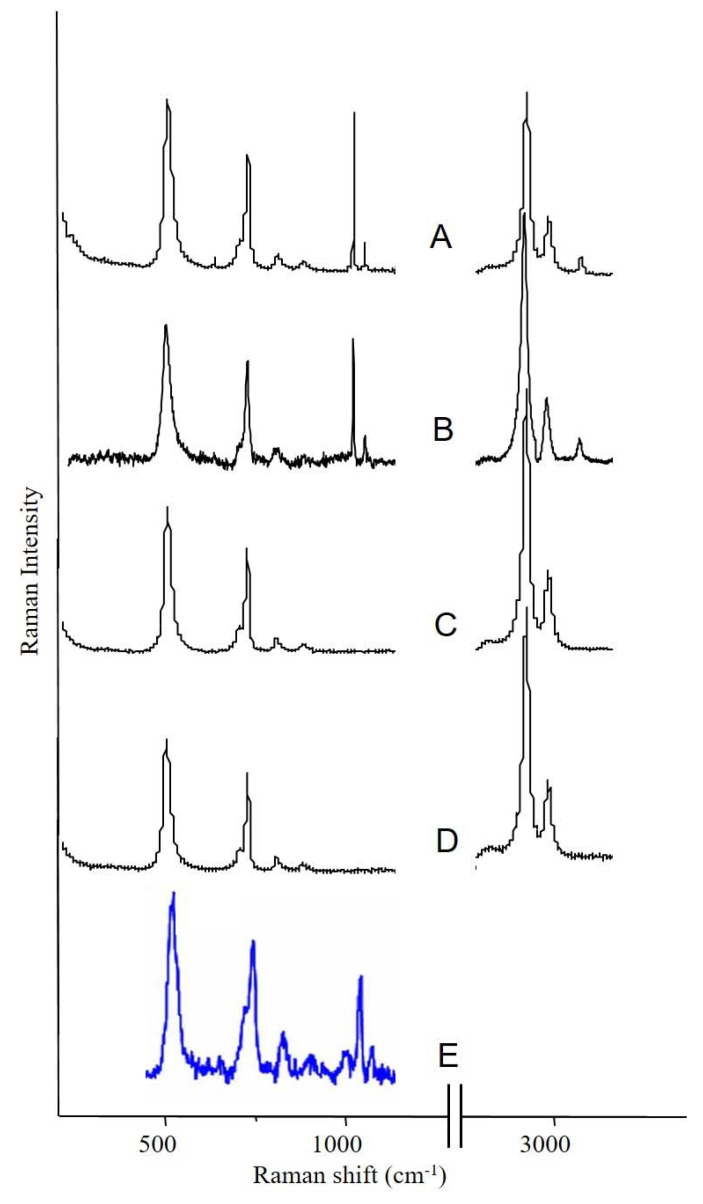
Raman spectroscopy analysis of the silicone breast implant: (**A**) spectrum of silicone migrated to fibrous tissue surrounding the implant; (**B**) spectrum of silicone obtained from the shell of the “explant”; (**C**) spectrum of silicone gel obtained from the inside of the “explant”; (**D**) reference PDMS material; (**E**) spectrum of the silicone obtained from the axillary lymph node.

**Figure 4:**
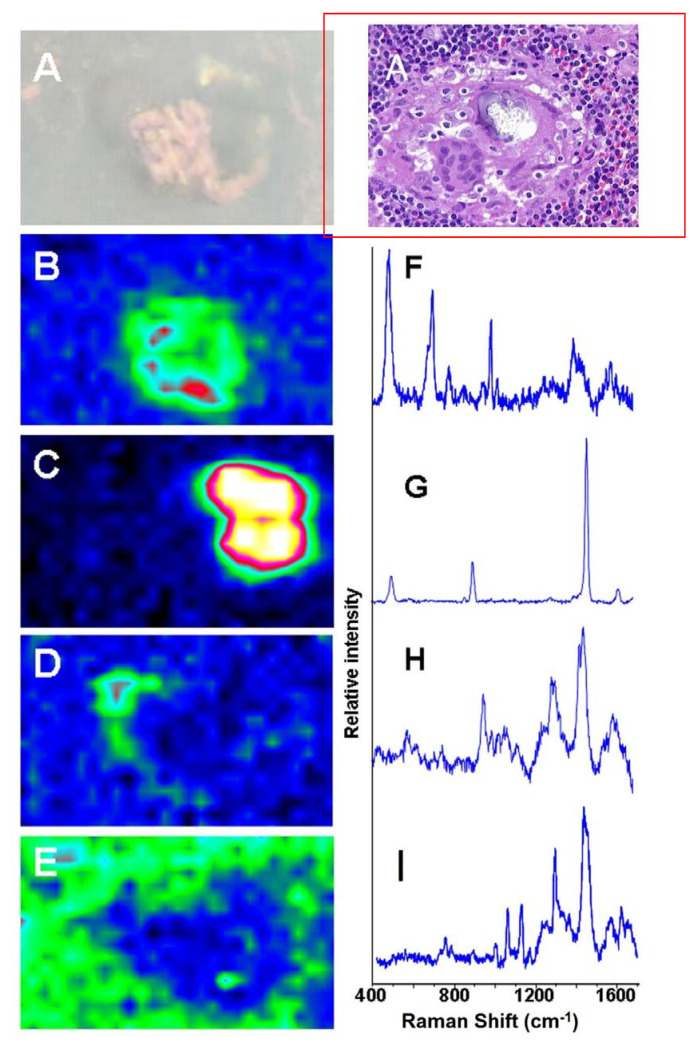
Raman spectroscopy generated images of silicone migrated to axillary lymph node: (**A**) white light and HE image; (**B**) Raman generated image based on the distribution of silicone shown in spectrum (**F**); (**C**) Raman image based on distribution of oxalate shown in spectrum (**G**); (**D**) Raman image based on the distribution of phosphate shown in spectrum (**H**); (**E**) Raman image of tissue around the migrated silicone; (**I**) Raman spectrum from the tissue surrounding the phenyl silicone and Schaumann body.

Figure 3 illustrates four different Raman microprobe studies. Trace A is the Raman spectrum of PDMS in the breast capsular connective tissue, while trace B is the Raman spectrum obtained from the surface of the “explant” shell. Trace C is the Raman spectrum of the silicone gel obtained from the inside of the “explant” (i.e., inner gel). Trace D (bottom trace) is the Raman spectrum of commercially obtained medical-grade silicone gel (Aldrich, Milwaukee, WI, USA) and is used as a reference for comparison. It is worth noting that the inner gel of the “explant” (C) showed the same spectrum as the commercially available (reference) PDMS (D), while the spectrum of PDMS that had migrated to the capsular breast tissue (A), and the spectrum from the outer surface of the explant capsule (B, implant shell), demonstrated new Raman lines at 621, 1031 (breathing vibration of the aromatic ring), 3056 (aromatic CH stretch), and one strong line at 1000 cm^−1^ (breathing vibration of the aromatic ring). The new bands are characteristic of the presence of aromatic groups bound to silicone-based compounds. This spectrum is consistent with the study by Keizers et al. in which the compound was identified as diphenyl silicone [21]. The presence of diphenyl silicone in the axillary lymph node section was also demonstrated employing CLRM (see Figure 4). The Raman spectroscopy generated images show the presence of diphenyl silicone (image B) surrounded by calcium phosphate (image C) and calcium phosphate mineral deposit (image D). The mineral deposits are identified histologically as Schaumann bodies. Schaumann irregular inclusions often resemble foreign-body giant cells. These inclusions have been described in the literature as calcified bodies which may be associated with foreign body granulomas. The Raman spectra (see Figure 4, traces G and H) unequivocally identify these Schaumann body inclusions as containing mineral deposition based on calcium oxalate and calcium phosphate minerals. The spectra of calcium oxalate and phosphate spectra are consistent with prior studies by Pestaner et al. and Calzolari et al. [26,27]. These findings suggest that not only inner silicone gel in the “highly cohesive” implant migrates to the surrounding tissues, but that axillary lymph nodes may also contain implant shell diphenyl silicone particles.

## 4. Discussion

Silicone leakage from traditional breast implants is frequent [4] but data on “highly cohesive” breast implants is scarce [28]. This type of implant was first introduced into the market in the early 1990s, claiming to reduce the amount of leakage of PDMS and thereby decreasing the possibility of local or systemic effects. A large follow-up study by Heden in 2012 showed a rupture rate of 1.7% in type 410 PDMS implants [29].

The relationship between silicone implants and systemic disease has been under investigation for many years [7]. Migration of silicone from implant “bleeds” typically follows the lymphatic pathway and is rarely found in tissue beyond the axillary lymph nodes [30]. Metastatic silicone granulomas have been described in patients who have had silicone oil injections for cosmetic reasons [31,32]. Initially, the risk of silicone leakage from highly cohesive implants was considered to be low, but case reports describing silicone spread from the reported generation of breast implants are rising in number [11,33,34,35,36,37]. In our case, the patient denied ever having silicone injections of any kind. After augmentation with the “highly cohesive” silicone gel implants, the woman described in this case study developed multifocal nodules consistent with sarcoidosis. PDMS was found in the fibrous capsules surrounding the implants and axillary lymph nodes both histologically and by CLRM spectroscopy. There is a possibility that surgical removal of the breast implants may have led to dissemination of silicone or other substances in the whole body, thereby worsening the patient symptoms.

There are more than 48 case reports of sarcoidosis of the breast [38,39,40,41,42,43,44,45,46]. There are two reports of patients with breast sarcoidosis who had silicone implants without exacerbation of their disease and who did not experience any unusual periprosthetic complications. One patient with silicone implants had progressive and non-responsive symptoms of sarcoidosis that did not remit until the implants were removed. One report describes a patient who presented with sarcoidosis of the skin, without systemic involvement [2,46,47,48]. A large study by Watad et al. in a cohort that contained over 24,000 women with breast implants matched with over 98,000 women without breast implants, showed a higher risk of developing sarcoidosis following silicone breast prosthesis implantation (OR 1.98) [49].

The etiology of sarcoidosis is unknown. It is a multisystem granulomatous disease that rarely involves the breast [50]. Immunologic abnormalities are the hallmark of sarcoidosis with the principal feature being non-caseating granulomas that somehow influence the function of the immune system by activating chemical mediators of inflammation or altering the function of lymphocytes [45,51].

It has been suggested that immunomodulation by foreign bodies such as silicone has a potential pathogenic role in the development of sarcoidosis [48]. Recently, silicone gel was shown to enhance both the humoral and the delayed-type hypersensitivity response of rats to bovine serum albumin, and therefore may also have a direct effect on macrophages with local release of various cytokines and subsequent recruitment and activation of lymphocytes [52]. The foreign body response to silicone or perhaps a direct adjuvant action of silicone, may contribute to systemic activation of macrophages and T-helper cells, serving as a stimulus in the progression of sarcoidosis [47].

## 5. Conclusions

This is the fifth in a series of individual case reports that links silicone breast implants to the development of sarcoidosis. There were non-caseating granulomas in four different body sites, an infectious work-up was negative, radiographic evidence of bilateral hilar lymphadenopathy, and an elevated ACE level and panuveitis of the left eye. This combination of findings is consistent with sarcoidosis. This is the first report where chemical spectroscopic mapping has been used to establish and identify the coexistence of Schaumann bodies, consisting of calcium oxalate and calcium phosphate minerals, in the presence of silicone. Whether the silicone in the tissues increased modulation of the immunologic response leading to the discovery of previously undiagnosed sarcoidosis, or whether it catalyzed the development of sarcoidosis is unknown.

## Data Availability

Not applicable.
